# CHREBP suppresses gastric cancer progression via the cyclin D1-Rb-E2F1 pathway

**DOI:** 10.1038/s41420-022-01079-1

**Published:** 2022-06-29

**Authors:** Jianming Zhang, Jing Zhang, Zhongmao Fu, Yuan Zhang, Zai Luo, Pengshan Zhang, Yitian Xu, Chen Huang

**Affiliations:** 1 Department of General Surgery, Shanghai General Hospital, Shanghai Jiaotong University School of Medicine, 100 Haining Road, Hongkou District, Shanghai, 200080 China; 2 Department of Thoracic Surgery, Shanghai Pulmonary Hospital, School of Medicine, Tongji University, Shanghai, 200433 China

**Keywords:** Gastric cancer, Prognostic markers, Transcriptomics

## Abstract

Accumulating evidence has demonstrated that carbohydrate response element binding protein (CHREBP) has a crucial function in tumor pathology. In this study, we found CHREBP downregulation in gastric cancer (GC) tissues, and CHREBP was determined to be an independent diagnostic marker of GC. The downregulation of CHREBP promoted cell proliferation and inhibited apoptosis. Moreover, the level of cyclin D1 was significantly correlated with CHREBP expression in GC and paracancerous normal samples. In addition, CHREBP transcriptionally inhibited cyclin D1 expression in GC cells. Tumor suppressor activity of CHREBP could be affected by the upregulation of cyclin D1. In summary, CHREBP was found to be an independent diagnostic marker of GC and to influence GC growth and apoptosis via targeting the cyclin D1-Rb-E2F1 pathway.

## Introduction

GC is the fifth most common malignant tumor and the third leading cause of cancer-associated deaths worldwide [1]. In 2020, the cancer-based statistics in the National Cancer Center of China indicated that the incidence and mortality of GC ranked the third and second, respectively [2]. Although the diagnosis and treatment of GC have markedly improved, approximately 80% of GC patients are already in advanced stages when they seek medical treatment, and the 5-year survival rate is < 20%, posing a serious risk to public health [3]. Therefore, in-depth exploration of the underlying molecular mechanisms is urgently needed, and identification of innovative targets for early diagnosis and treatment in GC would be highly valuable.

CHREBP, a transcription factor that is affiliated with the Mondo family [4], was first discovered in the nucleus of rat liver cells by Hasegawa in 1999 [5]. CHREBP is a macromolecular protein with a basic structure, namely, a helix-loop-helix/leucine zipper (bHLH/ZIP), and a molecular weight of 9.46 kilodaltons (kD) [6]. For potential functional domains, CHREBP contains a nuclear localization signal, a proline-rich stretch, a bHLH/ZIP, and a ZIP-like domain [7]. As previously research noted that CHREBP is essential for regulating glucose-responsive genes expression, but few studies on the tumorigenesis and development of GC have been conducted. Tong et al. found that CHREBP expression is closely relevant to the growth and proliferation of liver carcinomas [8]. Nevertheless, little is known regarding underlying mechanism of CHREBP in GC pathogenesis and development. Therefore, the function of CHREBP in GC growth is worthy of further in-depth assessment.

**Cyclin D1 functions** as a pivotal regulatory protein,which switches from G1 to S phase in cell cycle progression [9]. To date, numerous studies have described it as a proto-oncogene [10] and have confirmed its dysregulated expression in several types of cancer [11–16]. Uncontrolled cell proliferation and the tendency toward malignancy are likely due to its overexpression. The G1 phase suppressor protein Rb is phosphorylated by cyclin D1 after its dissociation from the E2F1 transcription factor. E2F1 further promotes genes transcription that thereby activate the cell cycle via inducing G1 to S phase [17]. However, underlying mechanism regulating cell cycle of GC cells remains elusive.

For current study, a low expression of CHREBP was detected in GC cells, and the regulation of CHREBP expression was found to influence both the proliferation and anti-apoptosis capabilities of cells. In particular, CHREBP was found to transcriptionally inhibit cyclin D1 expression in GC cells, and we identified a novel signaling pathway, the CHREBP-cyclin D1-Rb-E2F1 pathway, which critically regulates GC proliferation. Altogether, these results indicate that CHREBP could be a promising prognostic marker of GC.

## Results

### The level of CHREBP was low-expressed in GC samples, which was relevant to unfavourable prognosis

**Fig. 1:**
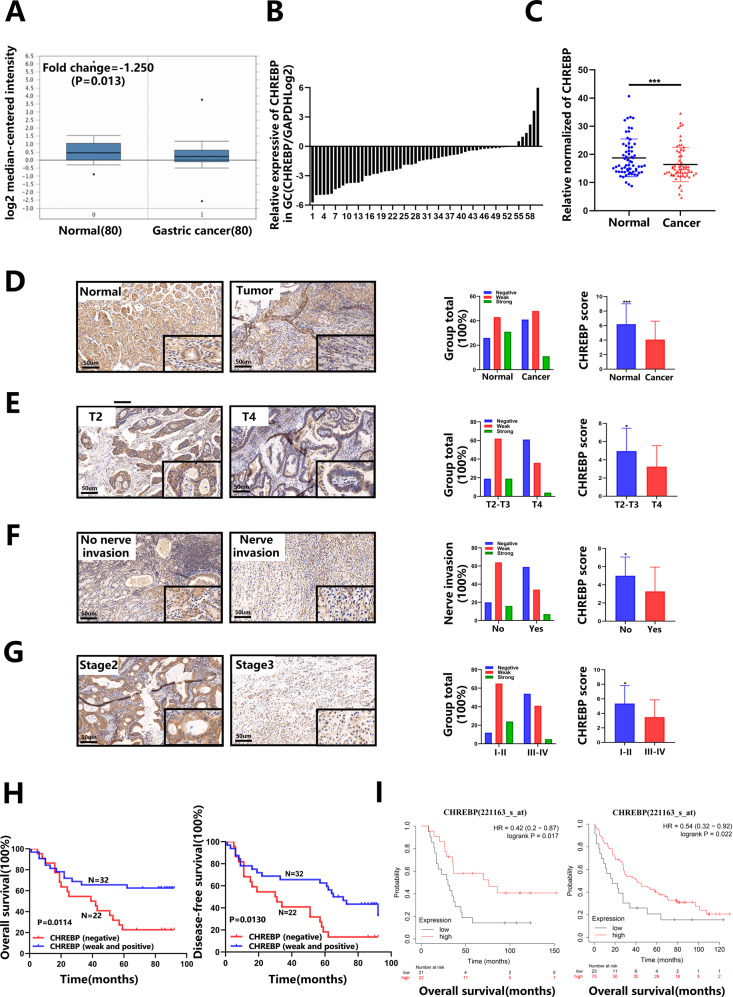
The level of CHREBP is downregulated in GC tissues and is associated with a poor prognosis. **A** Data from the Oncomine database showed that the level of CHREBP was decreased in GC in contrast with normal samples. **B**, **C** CHREBP expression in 60 GC and surrounding normal samples was examined by qRT–PCR. CHREBP expression was lower in 48 (80.00%) GC samples. **D** Representative pictures of CHREBP expression in GC and surrounding normal samples (scale bar: 50 μm) from TMAs. **E**–**G** Comparing the level of CHREBP between T2 and T3 vs. T4 (**E**, *P* = 0.004), No nerve invasion vs. Nerve invasion (**F**, *P* = 0.013), early TNM stage (stage I and II) vs. advanced TNM stage (stage III and IV) (**G**, *P* = 0.005). **H** Patients with lower CHREBP expression had worse OS (*P* = 0.0114) and DFS (*P* = 0.0130). **I** The data (GSE22377, left panel) and (GSE51105, right panel) from Kaplan–Meier plotter revealed that patients with decreased CHREBP expression had a shorter OS (*P* < 0.05).

The Oncomine database was searched to ascertain level of CHREBP in GC and surrounding normal samples, and it was revealed that level of CHREBP was markedly reduced in GC samples (Fig. 1A). To verify the low CHREBP expression in GC samples, qRT–PCR was conducted, and outcomes manifested that the total overall CHREBP expression was mostly lower in tumor than matched normal samples (Fig. 1B, C). The IHC results (containing 54 cancerous and noncancerous tissues) were concordant with the qRT–PCR results (Fig. 1D). The pathological parameters of the TMAs and 60 pairs of fresh-frozen tumor tissues are listed in Table 1 and Supplementary Table S3. CHREBP-positive dyeing was observed in both the nucleus and cytoplasm of GC cells, and CHREBP was positively stained in 32 of 54 cases (Fig. 1D, Table 1). Furthermore, the relationships between the pathological parameters and the level of CHREBP in GC samples were analyzed. The level of CHREBP was negatively relevant to T stage (*P* = 0.004), nerve invasion (*P* = 0.013), and TNM stage (*P* = 0.005) (Fig. 1E–G, Table 1). IHC outcomes of the TMAs manifested that CHREBP expression was downregulated in GC, which might exert an important influence on the development of GC.

**Table 1:** Correlation between the level of CHREBP and the pathological parameters of GC samples (*n* = 54).

			CHREBP expression level				
Parameters	Category	No.	Negative	Weakly positive	Strongly positive	*χ*^2^	*P*-value
Age at surgery (years old)	<60	15	5	8	2	0.494	0.781
	≥60	39	17	18	4		
Sex	Male	38	16	18	4	0.114	0.944
	Female	16	6	8	2		
T stage	T2-T3	26	5	16	5	11.150	**0.004**
	T4	28	17	10	1		
N stage	N0	11	2	8	1	3.686	0.158
	N1-N3	43	20	18	5		
TNM stage	I-II	17	2	11	4	10.805	**0.005**
	III-IV	37	20	15	2		
Nerve invasion	Yes	29	17	10	2	8.696	**0.013**
	N0	25	5	16	4		
Vessel invasion	Yes	29	15	12	2	3.514	0.173
	No	25	7	14	4		
Histological grade	Low	32	15	16	1	5.422	0.066
	Middle-high	22	7	10	5		
Tumor size (cm)	<5	26	10	11	5	3.637	0.162
	≥5	28	12	15	1		
Tumor site	Cardiac	13	6	7	0	3.537	0.171
	Non-cardiac	41	16	19	6		
Tumor and normal	Tumor	54	22	26	6	14.318	**0.001**
	Normal	54	14	23	17		

*p* values that are statistically significant are shown in bold.

Univariate analysis of 60 fresh-frozen GC tissues indicated that a lower CHREBP expression was relevant to a shorter OS (*P* = 0.032) and DFS (*P* = 0.036) (Supplementary Table S4). Similarly, the results of Kaplan–Meier method of TMAs manifested that the lower CHREBP expression was associated with a shorter OS (*P* = 0.0114) and DFS (*P* = 0.0130) (Fig. 1H). The results of the Kaplan-Meier Plotter database further confirmed that CHREBP expression was in negative correlation with OS (Fig. 1I). Additionally, N stage, TNM stage was also notably associated with OS; T stage, N stage, and TNM stage were markedly correlated with DFS (Table 2). Furthermore, multivariate survival analysis displayed that the level of CHREBP was associated with OS (hazard ratio (HR) = 0.414; 95% confidence interval (CI), 0.176–0.977; *P* = 0.044) but not with DFS. In the whole, these results demonstrate that CHREBP might function as an independent diagnostic marker for GC.

**Table 2:** The results of univariate and multivariate logistic regression analyses.

		OS				DFS			
Parameters	No.	Univariate analysis		Multivariate analysis		Univariate analysis		Multivariate analysis	
		*χ*^2^	*P*	HR (95%CI)	*P*	*χ*^2^	*P*	HR (95%CI)	*P*
Age		0.583	0.445			1.468	0.226		
<60	15								
≥60	39								
Sex		0.883	0.347			0.374	0.541		
Male	38								
Female	16								
T stage		1.372	0.242	0.510 (0.201–1.295)	0.157	5.714	**0.017**	0.837 (0.351–1.996)	0.689
T2-T3	26								
T4	28								
N stage		4.908	**0.027**	2.433 (0.332–17.831)	0.381	7.119	**0.008**	1.569 (0.257–9.584)	0.625
N0	11								
N1-N3	43								
TNM stage		6.903	**0.009**	2.587 (0.523–12.802)	0.244	11.647	**0.001**	3.246 (0.670–15.720)	0.144
I-II	17								
III-IV	37								
Nerve invasion		0.871	0.351			1.804	0.179		
Yes	29								
N0	25								
Vessel invasion		0.618	0.432			1.295	0.255		
Yes	29								
No	25								
Histological grade		0.233	0.629			0.000	0.990		
Low	32								
Middle-high	22								
Tumor size, cm		0.041	0.839			0.651	0.420		
<5	26								
≥5	28								
Tumor site		0.266	0.606			0.090	0.765		
Cardiac	13								
Non-cardiac	41								
CHREBP expression		6.403	**0.011**	0.414 (0.176–0.977)	**0.044**	7.058	**0.008**	0.509 (0.241–1.074)	0.076
Negative	22								
Weakly and strongly positive	32								

*p* values that are statistically significant are shown in bold.

**Fig. 2:**
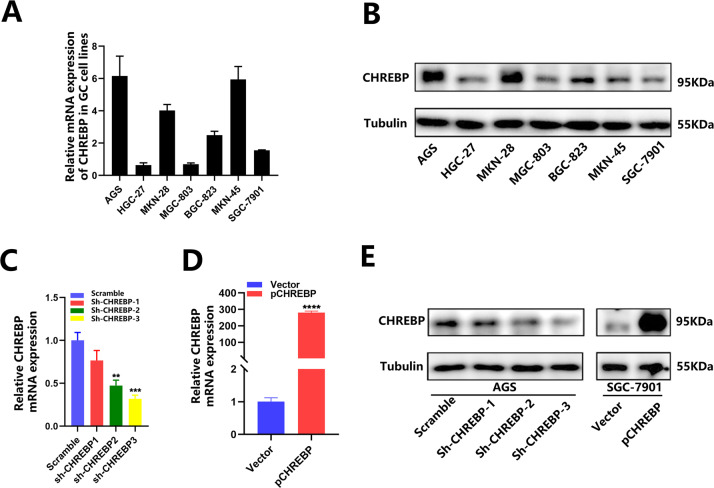
CHREBP expression of GC cell lines. **A**, **B** CHREBP expression of 7 GC cell lines was examined by qRT–PCR and WB. **C**–**E** CHREBP knockdown in AGS cells and CHREBP overexpression in SGC-7901 cells were detected by qRT–PCR and WB. Sh-CHREBP-3 was selected for the experiments.

To select the cell lines for subsequent assays, initial detection of CHREBP expression in GC cell lines by both qRT–PCR and WB (Fig. 2A, B), outcomes showed that CHREBP was relatively upregulated in AGS cells but relatively downregulated in SGC-7901 cells. Hence, AGS and SGC-7901 cells were chosen to establish stable cell lines for further functional assessment. Three different shRNAs were synthesized for AGS cells, while an overexpression lentivirus was designed for SGC-7901 cells, which presented lower and higher expression levels of CHREBP, respectively (Fig. 2C, D, E).

### CHREBP suppressed the GC cells proliferation in vitro

**Fig. 3:**
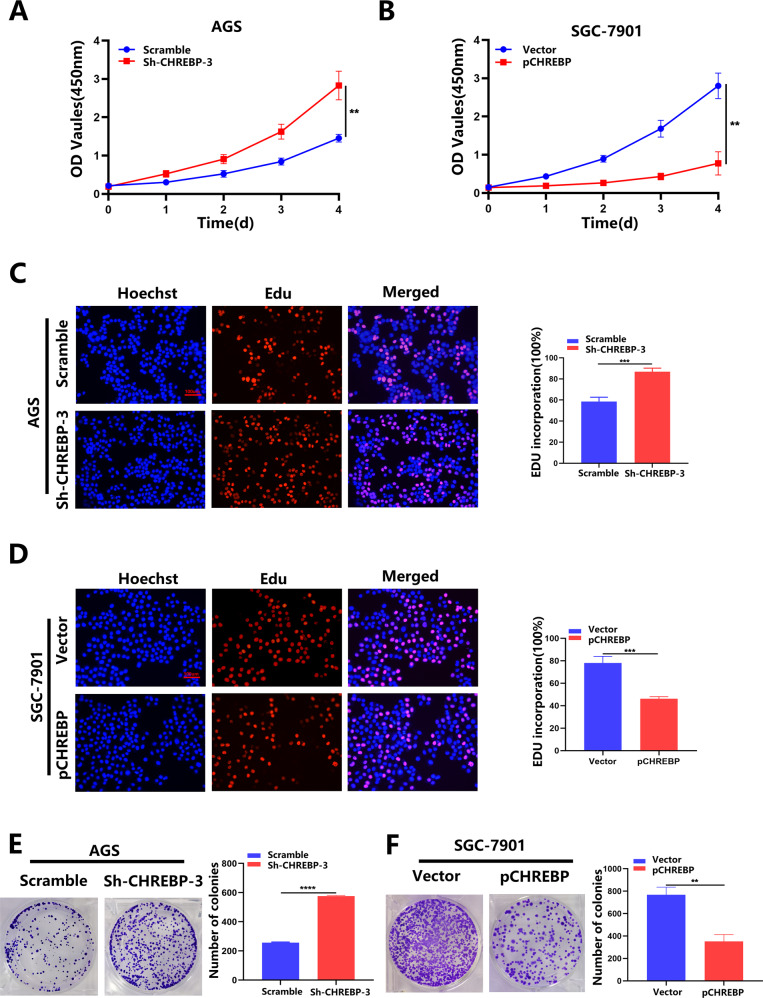
The effect of CHREBP on GC cell proliferation in vitro. **A**, **B** A CCK-8 assay was conducted to investigate the vitality of GC cells after CHREBP knockdown or overexpression. **C**, **D** An EdU assay was employed to testify proliferation of cells transfected with Sh-CHREBP or overexpressing CHREBP (scale bar: 100 μm). **E**, **F** A colony formation assay was conducted to examine the proliferation of Sh-CHREBP-transfected or CHREBP overexpression-transfected GC cells.

We attempted to assess the role of CHREBP in vitro. As shown in Fig. 3A, B, the proliferation of AGS cells was markedly elevated by downregulating the level of CHREBP, while upregulating the level of CHREBP remarkably suppressed the proliferation of SGC-7901 cells. The results of EdU assay confirmed that the number of EdU-positive AGS cells (proliferative cells) was increased in the CHREBP-downregulated group in comparison with the control group (Fig. 3C). In contrast, the CHREBP-overexpression group showed decreased in EdU-positive SGC-7901 cells (Fig. 3D). Similarly, the results of the colony formation assay also indicated that CHREBP knockdown in AGS cells significantly enhanced the colony formation ability, while the ability of colony formation in SGC-7901 cells was markedly attenuated by CHREBP overexpression (Fig. 3E, F).

**Fig. 4:**
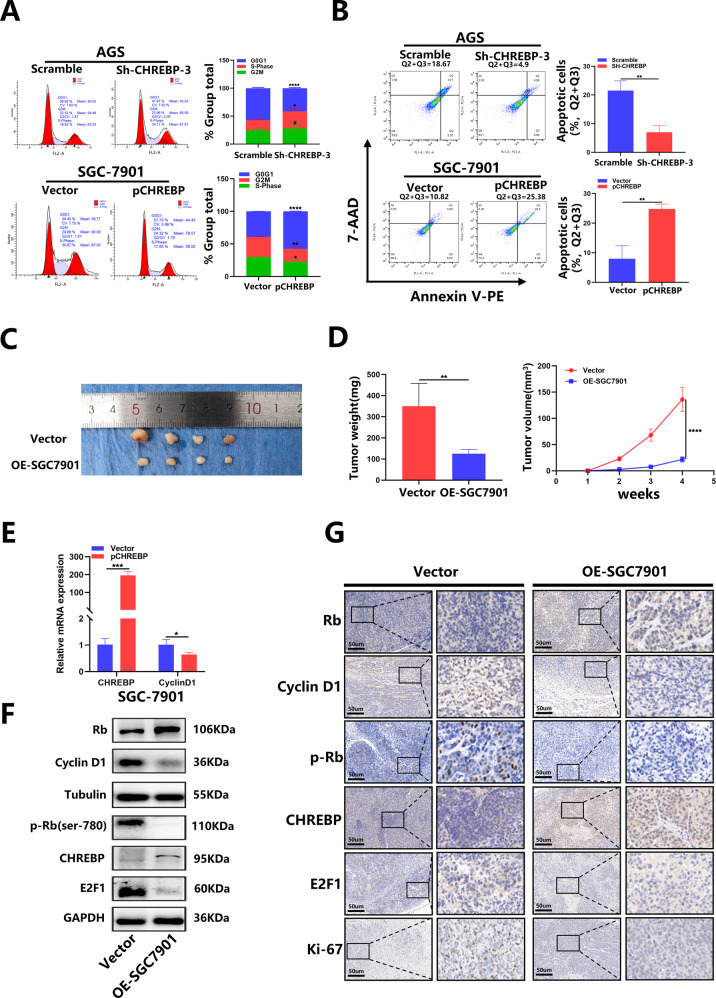
The effect of CHREBP on GC cell proliferation in vitro and in vivo. **A** Flow cytometry of AGS and SGC-7901 cells. **B** The rate of apoptosis of transfected cells was determined by flow cytometry (Q2 + Q3). **C** Images of tumors derived from different group transfected with empty vector and p-CHREBP of nude mice. **D** Tumors were assessed by their volume and average weight. E, Relative CHREBP and cyclin D1 expression were detected in subcutaneous tumor samples by qRT–PCR. **F** Relative Rb, cyclin D1, p-Rb (ser-780), CHREBP, and E2F1 expression were measured in subcutaneous tumor samples by WB. **G** Protein levels of Rb, cyclin D1, p-Rb (ser-780), CHREBP, E2F1, and Ki-67 in tumor samples were determined by IHC (scale bar: 50 μm). Data are shown as the mean ± SD in triplicate.

The results of flow cytometry revealed that in contrast with the control group, CHREBP knockdown markedly facilitated GC progression via the G1/S phase shift. Furthermore, CHREBP knockdown significantly inhibited apoptosis comparing with the control group. Conversely, CHREBP overexpression increased the ratio of cells in G0 and G1 phases while reduced the ratio in S phase and promoted apoptosis (Fig. 4A, B). These findings indicate that CHREBP functions as a tumor suppressor to restrain GC proliferation.

### CHREBP suppresses tumor growth in vivo

To explore CHREBP characteristics in vivo, a BALB/c nude mouse xenograft model of GC was established. SGC-7901/vector and SGC-7901-overexpressing (OE) cell suspensions were subcutaneously injected into male nude mice. As depicted in Fig. 4C, tumor growth was inhibited by CHREBP overexpression. The CHREBP overexpression group had a smaller tumor size and a decreased weight in contrast with the control group (Fig. 4D). In addition, qRT–PCR outcomes revealed that CHREBP expression was increased and cyclin D1 expression was downregulated in the OE-SGC7901 group (Fig. 4E). Western blot results displayed that the levels of CHREBP and Rb were upregulated and the levels of cyclin D1, P-Rb, and E2F1 were downregulated in the OE-SGC7901 group (Fig. 4F). Furthermore, the levels of cyclin D1, Rb, P-Rb, E2F1, and Ki-67 were examined by IHC, and the results revealed that the levels of CHREBP and Rb were increased and the levels of cyclin D1, P-Rb, E2F1, and Ki-67 were reduced in the OE-SGC7901 group in contrast with the control group (Fig. 4G). Taken together, these findings show that CHREBP exerts an inhibitory impact on tumor growth in vivo.

### CHREBP transcriptionally inhibited cyclin D1 expression in GC cells

**Fig. 5:**
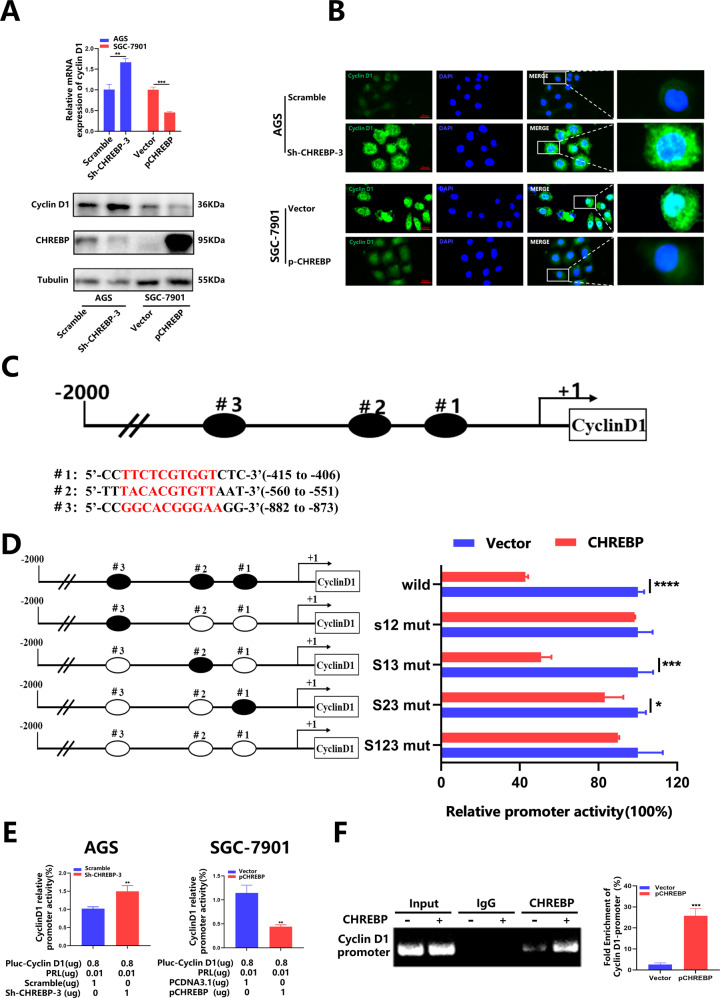
CHREBP downregulates cyclin D1 levels by binding to its promoters. **A** qRT–PCR and WB were conducted to detect CHREBP impact on cyclin D1 expression of GC cells. **B** IF assay was employed to estimate the expression and location of cyclin D1 in GC cells (scale bar: 20 μm). **C** The putative binding sites of CHREBP in the promoter region of cyclin D1. **D** Cyclin D1 promoters were transfected into SGC-7901 cells in triplicate with CHREBP expression or control vectors for 24 h. Cyclin D1 promoter activity was detected using a dual-luciferase reporter assay kit. Relative promoter activity in the treatment group was expressed as a percentage of that in the control group. **E** The effects of CHREBP on cyclin D1 promoter activity. A dual-luciferase reporter assay revealed that CHREBP could suppress cyclin D1 promoter activity in AGS and SGC-7901 cells. **F** CHIP assay and qRT–PCR showed that CHREBP could bind to cyclin D1 promoter region.

We changed the level of CHREBP in cells, and noticed that both the mRNA and protein levels of cyclin D1 were elevated when the level of CHREBP was downregulated in AGS cells; and vice versa the opposite trend was observed in SGC-7901 cells (Fig. 5A). Furthermore, we performed IF experiments to confirm the negative relation between the levels of CHREBP and cyclin D1 (Fig. 5B, Fig. S1A). The predicted sequence motifs from the JASPAR database are shown in Figure S1B. The data obtained from the JASPAR database revealed that CHREBP contained 3 binding sites in the promoter sequence of cyclin D1, including 5’-CCTTCTCGTGGTCTC-3’ (−415 to −406), 5’-TTTACACGTGTTAAT-3’ (−560 to −551), and 5’-CCGGCACGGGAAGG-3’ (−882 to −873) (Fig. 5C). According to the sites, we designed a full-length wild-type cyclin D1 promoter as well as mutants (S12-mut, S13-mut, S23-mut, and S123-mut). We then co-transfected the reporter plasmids with CHREBP-overexpression vectors or control vectors into SGC-7901 cells. Outcomes of the dual-luciferase reporter assay manifested that the mutation in the region from −560 to −551 bp, covering the #2 site (S13-mut), significantly increased the promoter activity of cyclin D1 that is inhibited by CHREBP (Fig. 5D). To further investigate whether CHREBP could regulate cyclin D1 promoter activity in GC cells, we co-transfected cells with CHREBP-overexpression or Sh-CHREBP-3 vectors. As displayed in Fig. 5E, CHREBP knockdown in AGS cells enhanced cyclin D1 promoter activity, and the SGC-7901 cells with increased expression of CHREBP showed the opposite effect. Finally, a CHIP assay was performed to determine whether CHREBP could act on the promoter region of cyclin D1, and CHREBP was indeed found to bind to the promoter region of cyclin D1 (Fig. 5F). Our findings revealed that CHREBP could bind to the #2 site (S13-mut) rather than other sites. These data indicate that CHREBP can transcriptionally inhibit cyclin D1 expression in GC cells.

To understand CHREBP role in the cyclin D1-mediated proliferative phenotype, enforced CHREBP expression in SGC-7901 cells was co-transfected with cyclin D1. As shown in Figure S2 (A, B), the level of cyclin D1 in SGC-7901 cells was found to be upregulated. As shown in Figure S2 (C–F), upregulation of CHREBP expression reduced the proliferation of GC cells. However, the overexpression of cyclin D1 partially reversed the suppressive impact on CHREBP bearing on GC proliferation, indicating that cyclin D1 participated in the proliferation of CHREBP-treated GC cells.

### The combination of CHREBP and cyclin D1 expression exhibited a valuable prognostic effect on GC

**Fig. 6:**
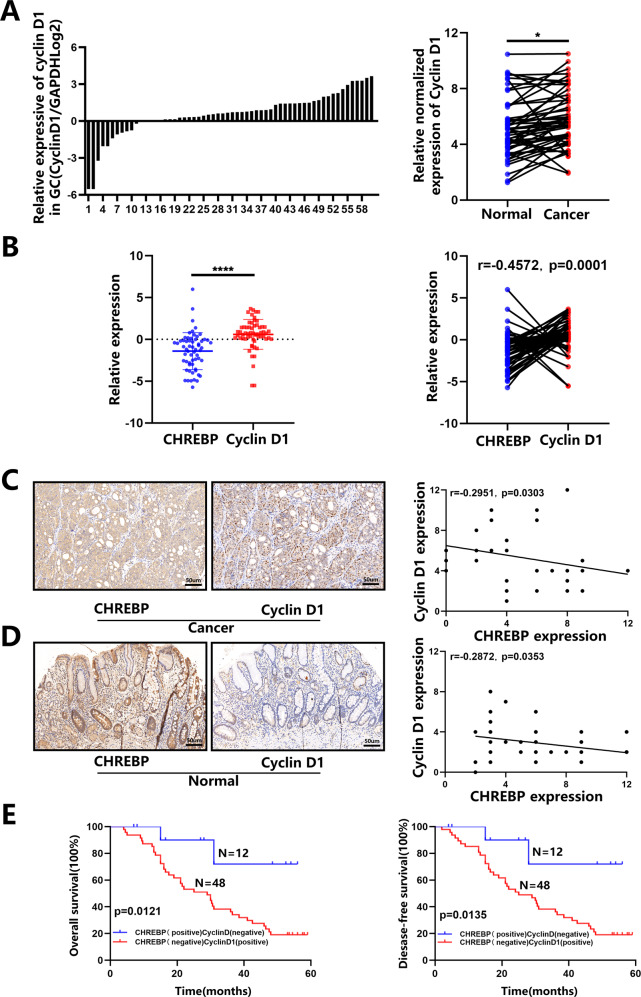
The combination of CHREBP and cyclin D1 levels is useful for predicting GC prognosis. **A** Relative mRNA level of cyclin D1 in 60 GC in contrast with that in matched normal para-carcinoma samples. **B** A negative relationship between mRNA levels of CHREBP and cyclin D1 in 60 GC samples (*r* = −0.4572, *P* = 0.0001). **C**, **D** A negative relationship between the levels of CHREBP and cyclin D1 in GC tissues (scale bar: 50 μm) (**C**; *r* = −0.2951, *P* = 0.0303) and adjacent normal tissues (**D**; *r* = −0.2872, *P* = 0.0353) was detected by IHC. **E** Prognostic values of CHREBP together with cyclin D1.

To further clarify the correlation between the levels of CHREBP and cyclin D1 in the same GC cases, we detected the levels of CHREBP and cyclin D1 in the same GC specimens, and it was found that cyclin D1 expression was mostly higher in the GC than in the control group (Fig. 6A). Further analysis showed a negative relationship between them in the GC in comparison with the control group, which was statistically significant (Fig. 6B). Moreover, Pearson’s correlation analysis demonstrated that CHREBP expression was weakly negatively correlated with the level of cyclin D1 in matched gastric mucosal and GC samples (Fig. 6C, D). Additionally, paired comparison analysis indicated that patients with low-expressed CHREBP (negative) and high-expressed cyclin D1 (positive) had notably worse OS and DFS than patients with high-expressed CHREBP (positive) and low-expressed cyclin D1 (negative) (Fig. 6E). Collectively, these results indicate that combination of CHREBP and cyclin D1 expression would be a significant diagnostic marker for GC.

### The CHREBP/cyclin D1 axis could regulate GC progression via the Rb/E2F1 pathway

**Fig. 7:**
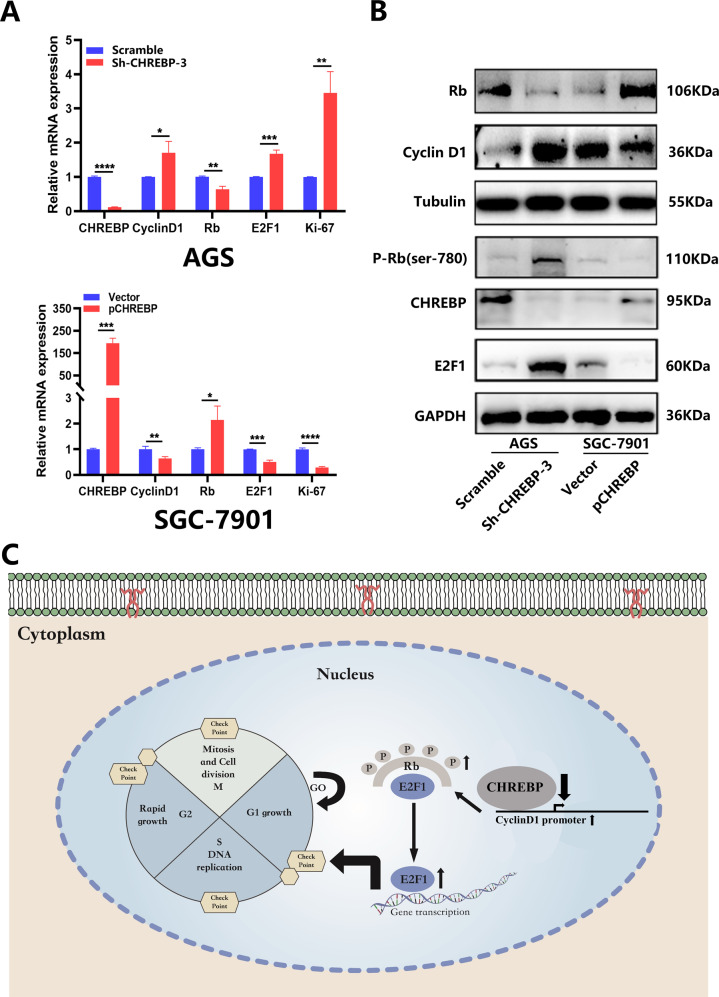
CHREBP regulates GC growth via the cyclin D1-Rb-E2F1 pathway. **A** Relative expression levels of CHREBP, cyclin D1, Rb, E2F1, and Ki-67 were detected in GC cells transfected with scramble, Sh-CHREBP, and an empty vector, p-CHREBP, as determined by qRT–PCR. **B** WB was used to detect the levels of Rb, cyclin D1, tubulin and phosphorylated Rb (P-Rb), CHREBP, E2F1, and GAPDH in GC cells transfected with scramble, Sh-CHREBP and an empty vector, p-CHREBP. **C** Concision model of CHREBP in regulating GC progression. The downregulated expression level of CHREBP specifically bound to cyclin D1 promoter, which increased the ability of cyclin D1 to inactivate Rb, leading to the release of E2F1, which could regulate the levels of genes involved in the cell cycle.

Collectively, the results showed that there was a negative interrelation between cyclin D1 and CHREBP expression of GC cells. As depicted in Fig. 7A, CHREBP knockdown significantly upregulated the mRNA levels of cyclin D1, E2F1, and Ki-67 while decreased the mRNA level of Rb. After overexpression of CHREBP in SGC-7901 cells, the mRNA levels of cyclin D1, E2F1, and Ki-67 were greatly decreased, whereas mRNA level of Rb was elevated. The same trend was found for other proteins (Fig. 7B). These results confirmed that the CHREBP/cyclin D1 axis regulated GC progression via the Rb/E2F1 pathway (Fig. 7C).

## Discussion

In the present study, a novel transcription factor, CHREBP, was assessed to determine its role in GC, and it was revealed that CHREBP suppresses GC progression by promoting cell apoptosis and inhibiting the cell cycle, thereby affecting tumor proliferation, and that it could serve as a significant prognostic biomarker. A reduction in CHREBP expression was found to upregulate the level of cyclin D1 through direct binding to its promoters, which ultimately led to the proposal of a novel signaling pathway that regulates GC proliferation: CHREBP/cyclin D1/Rb/E2F1.

Evidence is now accruing to confirm the significant function of CHREBP in tumor pathology and tumorigenesis [18]. CHREBP suppression in liver cancer and colorectal cancer tissues in vivo caused a reduction in adipogenesis and nucleotide synthesis, which also reduced cell proliferation and tumorigenicity [8]. Published research revealed that CHREBP expression is responsive to mitotic stimulation in immortalized hematopoietic cells [8]. In addition, CHREBP expression is noticeably higher in liver carcinoma tissues compared with the surrounding nontumor tissues in human liver samples [19]. An increased staining intensity of CHREBP has also been shown significantly positively associated with malignancy development of human breast carcinoma arrays [20]. Similar findings have been found in human metastatic prostate cancer tissues overexpressing CHREBP [21], which could partly explain the enhanced growth and invasiveness in castration-resistant prostate tumor cells. Notably, a TGFβ/Snail-dependent mechanism induced downregulation of CHREBP in epithelial-mesenchymal transition (EMT) during metastasis process of non-small-cell lung cancer [16]. However, loss of CHREBP in the induced mouse model presented an unimaginable antitumor effect and promoted the differentiation of leukemic primary cell [22]. The abovementioned findings suggest that the level of CHREBP follows tissue-specific spatiotemporal dynamics in malignancies.

For our study, CHREBP was found to act as a tumor suppressor. It was indicated that CHREBP expression was markedly decreased in GC samples according to the data obtained from the Oncomine database. Furthermore, the qRT–PCR outcomes indicated decreased CHREBP expression mainly in GC samples versus normal para-carcinoma samples. In addition, the results of IHC analysis of 54 cancerous and noncancerous tissues were consistent with those of qRT–PCR. CHREBP expression was also negatively significantly correlated with shorter OS and DFS in the survival analyses. Furthermore, in vitro experiments confirmed that the downregulation of CHREBP promoted the growth of GC cells and that the overexpression of CHREBP exerted inhibitory effects, which could be further proven by an in vivo tumor xenograft assay.

Mechanistically, CHIP-seq analysis of CHREBP gene binding in mouse liver and HepG2 cells indicates that the role of CHREBP in tumorigenesis has augmented from metabolic reprogramming to cell cycle control [23, 24]. The regulatory effect of CHREBP on cell cycle-related genes has been reported in benign β-cell growth and malignancy activity of prostate carcinoma, but correlational study involving CHREBP binding directly to the promoter regions of these genes is still lacking [17, 25]. A study suggested that CHREBP interacted with confirmed co-carcinogenic factor, including the c-Myc or HIF, which might be involved in the induction of the cell cycle or tumor microenvironment [26].

Cells have developed detection mechanisms to ensure DNA replication and chromosome allocation during the cell cycle that have been preserved over long-term evolution, which are typically called checkpoints or restriction points. There are three checkpoints of cell cycle, namely, G1/S phase, G2/M phase, and spindle assembly checkpoint [27]. Cyclin D1 is a proto-oncogene located on chromosome 11q13 [28, 29], and it is a key protein that regulates the G1 phase in cell cycle. Importantly, cyclin D1 up-regulation induces DNA repair/ replication process, which is relevant to genetic instability via promoting the frequency of gene deficiency or gene amplification [30]. E2F1 promotes cell cycle progression and may contribute to GC progression by increasing levels of the genes involved in the cell cycle.

Transcription factor activity could be transformed through a variety of direct mechanisms, which were demonstrated in multiple cancer types, chromosomal translocations, gene amplification or deletion, point mutations, and altered expression included [31, 32]. As previously noted in our research that direct binding to the vimentin promoter domain of SIX1 can increase the level of vimentin at the transcriptional level, thereby facilitating GC cells migrated and invasive ability [33]. KLF4 was discovered to be downregulated in GC and pancreatic tumor. KLF4 knockdown was shown to promote EMT and metastasis in GC by targeting PODXL. Direct binding of KLF4 to the promoter region of caveolin-1 and reduced the level of caveolin-1 to inhibit EMT and metastasis in pancreatic cancer [34].

CHIP assay manifested that CHREBP direct bound to the cyclin D1 promoter, and dual-luciferase reporter assay strongly proved that CHREBP knockdown might enhance cyclin D1 promoter activity and transcriptionally upregulate the level of cyclin D1, which could be participated in carcinogenic mechanism underlying CHREBP-induced tumorigenesis. We still found that CHREBP knockdown immensely upregulated mRNA levels of cyclin D1, E2F1, and Ki-67 while negatively regulating the mRNA level of Rb. However, CHREBP overexpression led to the opposite results. Altogether, these results confirmed that the CHREBP/cyclin D1 axis could regulate GC progression via the Rb/E2F1 pathway, the activation of which has been elaborated to promote G1/S transition and enhance tumor cells proliferation [35].

To sum up, our study indicated that CHREBP could be an vital tumor suppressor gene for GC, as its expression level was significantly decreased in GC cells. A reduction in CHREBP expression contributed to GC growth in vitro and in vivo by transcriptionally upregulating cyclin D1 expression. An increased cyclin D1 expression further inactivated the tumor suppressor gene Rb and released the transcription factor E2F1. Importantly, we not only confirmed that CHREBP could be a valuable prognostic marker for GC but also revealed the significance of the CHREBP/cyclin D1/Rb/E2F1 pathway in GC development, which might assist clinicians in more effectively treating GC patients.

## Methods

### Specimen collection and cell culture

From February 2015 to October 2017, a total of 60 specimens were gained from GC patients undergoing radical gastrectomy at Shanghai General Hospital (Shanghai, China) and stably reserved at −80 °C for subsequent RNA assay. A total of 57 wax specimens from GC patients who underwent radical surgery were collected from March 2013 to January 2014. After a series of immunohistochemical procedures, 57 cases of GC and para-cancerous formalin-fixed paraffin-embedded blocks were reviewed and diagnosed by two pathologists (with more than 10 years of experience), after which 3 pairs of non-mucosal wax blocks were excluded. Finally, 54 paired GC samples were identified for inclusion and subjected to tissue microarrays (TMAs). The subject was with approval of the Ethics Committee of the Shanghai General Hospital. Seven human GC cell lines were adopted at follow-up investigation: HGC-27, MKN-28, MKN-45, MGC-803, BGC-823, AGS, and SGC-7901. All cell lines were obtained from the Cell Center of the Chinese Academy of Sciences (Institute of Life Sciences, Shanghai, China), which were cultured by RPMI-1640 medium (Gibco, USA) added in 10% fetal bovine serum (Gibco, USA), 1% penicillin and streptomycin (Gibco, USA) included, as previously described [36].

### Vector construction and transfection

Full-length CHREBP was synthesized to generate CHREBP-overexpressing lentiviral vectors (pLVX-CHREBP) to evaluate its role in GC progression. ShCHREBPs lentiviral vectors was employed for CHREBP knockdown (OBiO Technology Co., Ltd., Shanghai, China). The targeted sequences for particular shRNAs (shCHREBP#1–3) were synthesize indicated below: Sh-CHREBP-1: 5′- GGCGCATCTACTACAAGAA-3′; Sh-CHREBP-2: 5′-GGCCTGGTATATCCAGTAT-3′; and Sh-CHREBP-3: 5′-CAAGCCTGGATGACTTCAT-3′. In addition, lentivirus was transfected into AGS and SGC‑7901 cells, and Lipofectamine 2000 (Invitrogen, USA) was adopted to conduct plasmid DNA transfection in accordance with the manufacturer’s protocols. Five microliters plasmid to be transfected was aspirated, and 5 microliters transfection reagent was added to 250 µl Opti-MEM medium (Thermo Fisher Scientific, USA) without serum and antibiotic. The solution was mixed gently and incubated for 20 min at room temperature prior to transfection. Cells were harvested 48 h after transfection for subsequent analysis, as previously described [37].

### Flow cytometry

Cells were harvested, fixed, and then stained with 0.5 mL of propidium iodide/RNase staining buffer (BD Biosciences, USA) in the dark for half an hour. A flow cytometer (BD Biosciences, USA) was used to analyze the percentages of the respective parts of the cells. GC cells at the logarithmic growth stage were taken and digested by trypsin into a single-cell suspension. After suction and purification, 10 μL of 7-AAD and 5 μL of Annexin V-PE (BD Biosciences, USA) were added to each tube successively. Then, the cell suspension was gently mixed, followed by incubation for 15–30 min without light. Cell apoptosis rate was calculated as previously specified [38].

### Construction of the tumor xenograft animal model

BALB/c nude mice were injected with SGC-7901 cells (5 × 10^6^ cells/100 μl) to establish a tumor xenograft animal model. Tumor volumes were measured once a week following the formula: volume = (length × width^2^)/2 [39]. All mice were subject to sacrifice after the completion of the experiments according to ethical standards.

### RNA extraction and quantitative reverse transcription-polymerase chain reaction (qRT–PCR)

Total RNA from GC samples was extracted using TRIzol (TaKaRa, Tokyo, Japan) methods, then reverse transcribed into cDNA using the isolated RNA (500 ng). Then, qRT–PCR was conducted adopting the SYBR Premix Ex Taq kit (Takara, Tokyo, Japan) with cDNA acting as the amplified template. Gene expression was analyzed by the 2^-ΔΔCt^ method. The primer sequences for qRT–PCR were summarized in Supplementary Table S1. All experiments were carried out with three replicates.

### Western blot (WB) analysis

Total protein extraction was performed with RIPA lysis supplemented with 1% PMSF (Beyotime, China), then protein quantification was determined by the BCA kit (Beyotime, China) method. Proteins were electrophoresed into a 10% SDS–PAGE gel (New Cell & Molecular Biotech Co., Ltd, Suzhou, China) and then transferred into a PVDF membrane (Millipore, USA), which was blocked in 5% nonfat milk (Yili Co., Ltd, China) and afterwards incubated with the corresponding antibodies. The list of antibodies used was presented in Supplementary Table S2. Tubulin (1:1000, #2146, Cell Signaling Technology, USA) and GAPDH (1:1000, #97166, Cell Signaling Technology, USA) were used for standardized protein. Immunoblotting image was displayed in ECL reagent (Millipore, USA), and images were processed by Image Lab Software (Bio–Rad Laboratories Inc., Hercules, USA).

### Cell proliferation assay

6-well plates were seeded with 1000 cells/well lasting 2 weeks to conduct colony formation. Then, they were washed, fixed, stained, afterwards the colonies were calculated and analyzed. Cell counting kit-8 (CCK-8) assay was proceeded as previously noted [36]. In the EdU assay, 24-well plates were employed to culture cells overnight, cells were pulsed by 10 μM EdU lasting 2 h. Apollo and Hoechst 33342 staining solutions were prepared for the staining of cell nuclei for at least 30 min. Images were photographed via fluorescence microscope (Leica, Wetzlar, Germany). All experiments were conducted in triplicate.

### Immunohistochemistry (IHC)

Samples were fixed, sectioned and incubated with antibodies. The CHREBP (1:100, #NB400-135, Novus Biologicals, Germany) and cyclin D1 (1:250, #55506, Cell Signaling Technology, USA) intensity proportions were categorized indicated below: 0–10% (0 points), 10–25% (1 points), 25-50% (2 points), 50-75% (3 points), and >75% (4 points). Staining intensity scores were evaluated as following: 0 points for no staining, 1 point for blue, 2 points for brown, and 3 points for dark brown. Final score was multiplying results between the staining intensity and proportion. In this experiment, 0–3 points were judged as negative, 4–7 points were judged as weakly positive, and 8–12 points were judged as strongly positive.

### Immunofluorescence (IF) assay

AGS and SGC-7901 cells were cultured on a confocal laser dish, which were then washed, fixed, permeabilized. Afterwards it was incubated with anti-CHREBP (1:100, **#**NB400-135, Novus Biologicals, Germany) and anti-cyclin D1 (1:250, **#**55506, Cell Signaling Technology, USA) antibodies at 4 °C for 24 h. Samples were then blocked, washed, and incubated with a FITC-conjugated goat anti-rabbit or Cy3-conjugated goat anti-mouse IgG antibody (Thermo Fisher Scientific, Waltham, MA, USA). In addition, DAPI (1:1000, RiboBio, China) was employed to stain cell nuclei for 3 min. TCS SP8 confocal microscope (Leica, Wetzlar, Germany) was utilized to microscopically analyze cells.

### Dual-luciferase reporter assay

The pGL4.27-cyclin D1-wild promoter or mutant type (termed S12mut, S13mut, S23mut, S123mut) was transfected with pCHREBP or the control vector into the target cells. Cyclin D1 promoter activity was normalized to Renilla luciferase reporter, which acted as internal reference in accordance with the manufacturer’s protocols for the Dual Luciferase Assay Kit (Promega, USA). The luciferase activity results were assessed by a spectrophotometer (Molecular Devices, USA). The ratio of firefly luciferase/Renilla activity was conducted to calculate CHREBP impact on the luciferase reporter.

### Chromatin immunoprecipitation (ChIP) assay

SGC-7901 cells (4 × 10^6^) stably transfected with CHREBP or vector were well cultured for ChIP via ChIP assay kit (Millipore, USA) in accordance with the manufacturer’s instructions. Then, 270 µl of formaldehyde (Sangon Biotech, China) was directly added to a petri dish containing 10 ml of medium for histone and DNA crosslinking at an ultimate concentration of 1% and incubated for 10 min at 37 °C. After crosslinking, cells were resuspended in 200 µL of SDS lysis buffer (Sangon Biotech, China) and incubated on ice for 10 min to quench reaction. Then, the cells were lysed and disrupted by sonication on ice until the DNA was sheared into fragments between 200 and 1000 base pairs in size. The sonicated samples were added to Protein A/G Agarose beads before incubation with 2 μg anti-CHREBP overnight at 4 °C for antigen-antibody binding. The final DNA fragments containing the cyclin D1 promoter were amplified using PCR with the primers 5′- ATTCTGCCGGCTTGGATATGGG-3′ (forward) and 5′- TTTCTCCCCGCCAGGGAGA-3′ (reverse).

### Statistical analysis

The data were analyzed by SPSS 25.0 software (IBM, Armonk, NY, USA). Student’s *t*-test or the Mann–Whitney U test were utilized for the paired and unpaired continuous variables. Fisher’s exact test or the χ^2^ test were conducted to analyze categorical variables. Spearman’s correlation test was employed to evaluate the relationship between CHREBP and cyclin D1 expression. Overall survival (OS) and disease-free survival (DFS) were plotted via the Kaplan–Meier method and the log-rank test, respectively. *P*-value less than 0.05 was considered statistically significant.

## Supplementary information

SUPPLEMENTAL MATERIAL

Original Data File

Figure S1

Figure S2

## Data Availability

The data generated or analyzed in this study were included in the published article and its additional files.
