# Supplementary material for: CHREBP suppresses gastric cancer progression via the cyclin D1-Rb-E2F1 pathway
Source: Cell Death Discov. 2022 Jun 29;8:300. doi: 10.1038/s41420-022-01079-1 (PMC9243070; doi:10.1038/s41420-022-01079-1)
Supplement: Supplementary file 1 — SUPPLEMENTAL MATERIAL [file 41420_2022_1079_MOESM1_ESM.docx]

**Fig. S1. The cellular** [**colocalization**](javascript:;) **of CHREBP and cyclin D1.** A, Immunofluorescence assay was performed to assess the cellular location of CHREBP (green) and cyclin D1 (red) in cells (scale bar: 25 μm). B, The predicted sequence motifs downloaded from the JASPAR database.

**Fig. S2. Rescue assay results by overexpressing cyclin D1 on the basis of CHREBP overexpression.** A, B, In SGC-7901 cells, upregulation of cyclin D1 was conducted on the basis of the upregulation of CHREBP. C, A CCK-8 assay was used to determine the viability of GC cells. D, An EdU assay was employed to verify the proliferation of cells transfected with pCHREBP+vector and pCHREBP+pCyclin D1. E, A colony formation assay was conducted to detect the proliferation of pCHREBP+vector-transfected or pCHREBP+pCyclin D1-transfected GC cells. F, Flow cytometry of SGC-7901 cells. G, The rate of apoptosis of transfected cells was determined by flow cytometry (Q2 + Q3).

**Supplement Table S1: The primers for experiments were listed as following**

| **primes** | **primes sequences** |
| --- | --- |
| CHREBP forward | 5′-GCGTTTTGACCAGATGCGAGAC-3′ |
| CHREBP reverse | 5′-CGTTGAAGGACTCAAACAGAGGC-3′ |
| CyclinD1 forward | 5′-TCTACACCGACAACTCCATCCG-3′ |
| CyclinD1 reverse | 5′-TCTGGCATTTTGGAGAGGAAGTG-3′ |
| Rb forward | 5′-CAGAAGGTCTGCCAACACCAAC-3′ |
| Rb reverse | 5′-TTGAGCACACGGTCGCTGTTAC-3′ |
| E2f1 forward | 5′-GGACCTGGAAACTGACCATCAG-3′ |
| E2f1 reverse | 5′-CAGTGAGGTCTCATAGCGTGAC-3′ |
| Ki-67 forward | 5′-GAAAGAGTGGCAACCTGCCTTC-3′ |
| Ki-67 reverse | 5′-GCACCAAGTTTTACTACATCTGCC-3′ |
| GAPDH forward | 5′ -GTCTCCTCTGACTTCAACAGCG-3′ |
| GAPDH forward | 5′-ACCACCCTGTTGCTGTAGCCAA-3′ |

**Supplement Table S2: The list of antibodies used for experiments**

| **Gene Symbol** | **Catalog number** | **Dilution ratio** | **Antibody Company** |
| --- | --- | --- | --- |
| CHREBP | #NB400-135 | 1:1000 | Novus biologicals |
| CyclinD1 | #55506 | 1:1000 | Cell Signaling Technology, |
| Rb | #D151675 | 1:1000 | sangon biotech |
| P-Rb | #3590 | 1:1000 | Cell Signaling Technology |
| E2F1 | #66515-1-Ig | 1:2000 | Proteintech |
| Tubulin | #2146 | 1:1000 | Cell Signaling Technology |
| GAPDH | #97166 | 1:1000 | Cell Signaling Technology |

**Table S3 Correlation between expression of CHREBP and clinicopathological features in 60 GC fresh-frozen Tissues**

|  |  | | |  | **CHREBP expression** | |  |  |
| --- | --- | --- | --- | --- | --- | --- | --- | --- |
| **Parameters** | | **Category** | **No.** | | **Low** | **High** | **χ^２^** | **P value** |
| Age at surgery (years old) | | <60  ≥60 | 14  46 | | 14  34 | 0  12 | 3.080 | 0.079 |
| Sex | | Male  Female | 46  14 | | 39  9 | 7  5 | 1.683 | 0.195 |
| T stage | | T1-T2  T3-T4 | 12  48 | | 6  42 | 6  6 | 6.257 | **0.012** |
| N stage | | N0  N1-N3 | 18  42 | | 12  36 | 6  6 | 1.791 | 0.181 |
| TNM stage | | I-II  III-IV | 23  37 | | 15  33 | 8  4 | 5.094 | **0.024** |
| Nerve invasion | | Yes  N0 | 38  22 | | 34  14 | 4  8 | 4.311 | **0.038** |
| Vessel invasion | | Yes  No | 43  17 | | 37  11 | 6  6 | 1.049 | 0.306 |
| Histological grade | | Low  Middle-high | 43 17 | | 36  12 | 7  5 | 0.621 | 0.431 |
| Tumor size，cm | | <5  ≥5 | 27  33 | | 23  25 | 4  8 | 0.825 | 0.364 |
| Tumor site | | Cardiac  Non-cardiac | 14  46 | | 12  36 | 2  10 | 0.052 | 0.819 |

**Table S4:** **The results of univariate and multivariate logistic regression analyses in 60 GC fresh-frozen Tissues**

|  |  | **OS** | | | | **DFS** | | | | | | | | | |  |  |
| --- | --- | --- | --- | --- | --- | --- | --- | --- | --- | --- | --- | --- | --- | --- | --- | --- | --- |
| **Parameters** | **No.** | **Multivariate** | | **Univariate analysis** | | | | **Multivariate Univariate analysis** | | | | | | | |  |  |
|  |  | **χ^2^** | **P** | **HR（95%CI）** | **P** | | **χ^2^** | | **P** | **HR（95%CI）** | | **P** | | | |  |  |
| Age  <60  ≥60 | 14  46 | 0.029 | 0.864 |  |  | | 0.028 | | 0.868 |  | |  | | | |  |  |
| Sex  Male  Female | 46  14 | 0.389 | 0.533 |  |  | | 0.344 | | 0.557 |  | |  | | | |  |  |
| T stage  T1-T2  T3-T4 | 12  48 | 0.206 | 0.650 |  |  | | 0.256 | | 0.613 |  | |  | | | |  |  |
| N stage  N0  N1-N3 | 18  42 | 0.493 | 0.483 |  |  | | 0.469 | | 0.493 |  | |  | | | |  |  |
| TNM stage  I-II  III-IV | 23  37 | 0.828 | 0.363 |  |  | | 0.937 | | 0.333 |  | |  | | | |  |  |
| Nerve invasion  Yes  N0 | 38  22 | 0.295 | 0.587 |  |  | | 0.280 | | 0.597 |  | |  | | | |  |  |
| Vessel invasion  Yes  No | 43  17 | 0.021 | 0.885 |  |  | | 0.030 | | 0.863 |  | |  | | | |  |  |
| Histological grade  Low  Middle-high | 43  17 | 1.235 | 0.266 |  |  | | 1.141 | | 0.285 |  | |  | | | |  |  |
| Tumor size，cm  <5  ≥5 | 27  33 | 9.011 | **0.003** | 0.415（0.221-0.781） | **0.006** | | 8.504 | | **0.004** | 0.423（0.225-0.797） | | | 0.008 | | | | |
| Tumor site  Cardiac  Non-cardiac | 14  46 | 0.601 | 0.438 |  |  | | 0.722 | | 0.395 |  |  | | | |  |  |  |
| CHREBP  Low  High | 48  12 | 4.600 | **0.032** | 0.325（0.100-1.057） | 0.062 | | 4.381 | | **0.036** | 0.332（0.102-1.078） | | | | 0.066 | | |  |
