# Supplementary material for: CHREBP suppresses gastric cancer progression via the cyclin D1-Rb-E2F1 pathway
Source: Cell Death Discov. 2022 Jun 29;8:300. doi: 10.1038/s41420-022-01079-1 (PMC9243070; doi:10.1038/s41420-022-01079-1)
Supplement: Supplementary file 2 — Original Data File [file 41420_2022_1079_MOESM2_ESM.pdf]

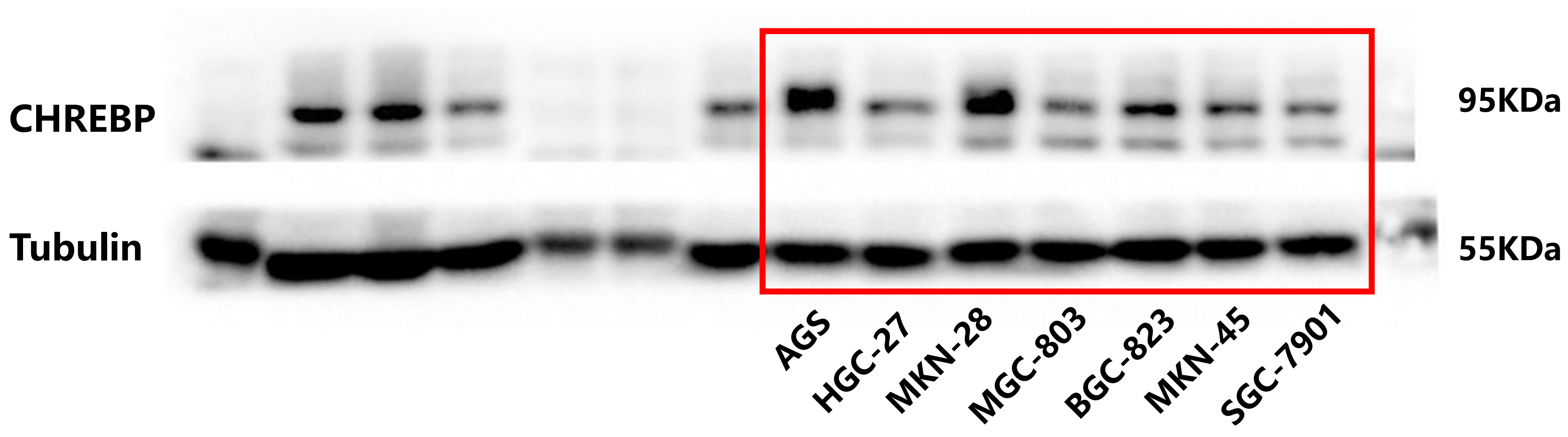
CHREBP
Tubulin
95KDa
55KDa
AGS
HGC-27
MKN-28
MGC-803
BGC-823
MKN-45
SGC-7901

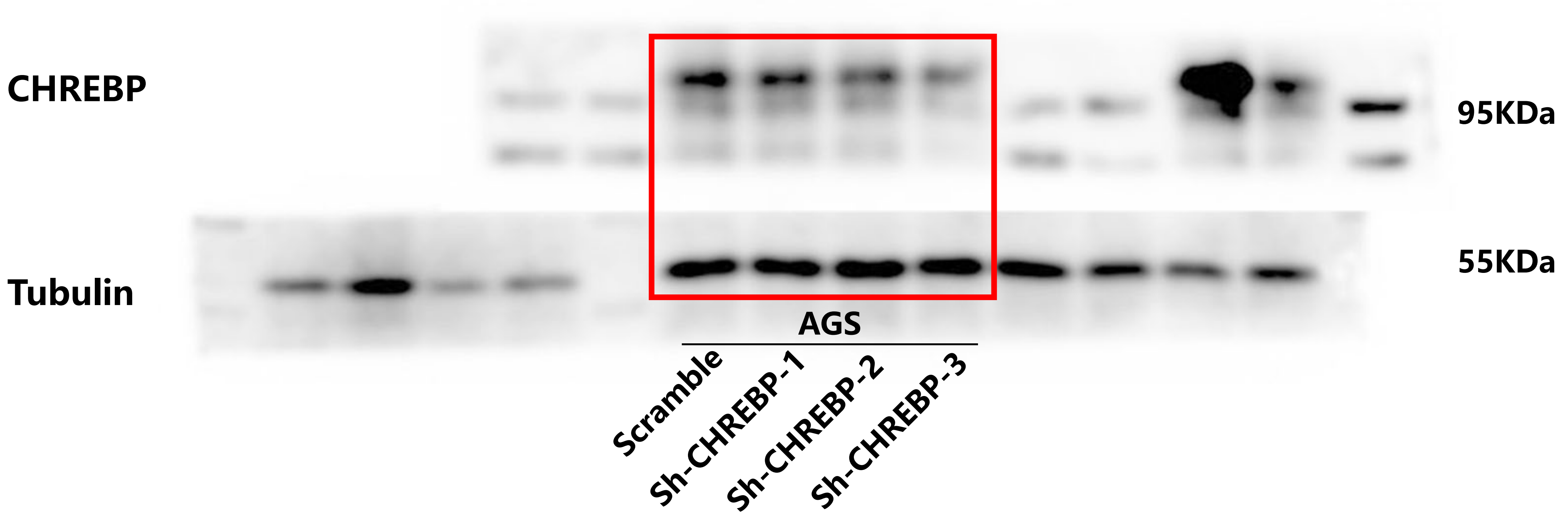
CHREBP
95KDa
55KDa
Tubulin
AGS
Scramble
Sh-CHREBP-1
Sh-CHREBP-2
Sh-CHREBP-3

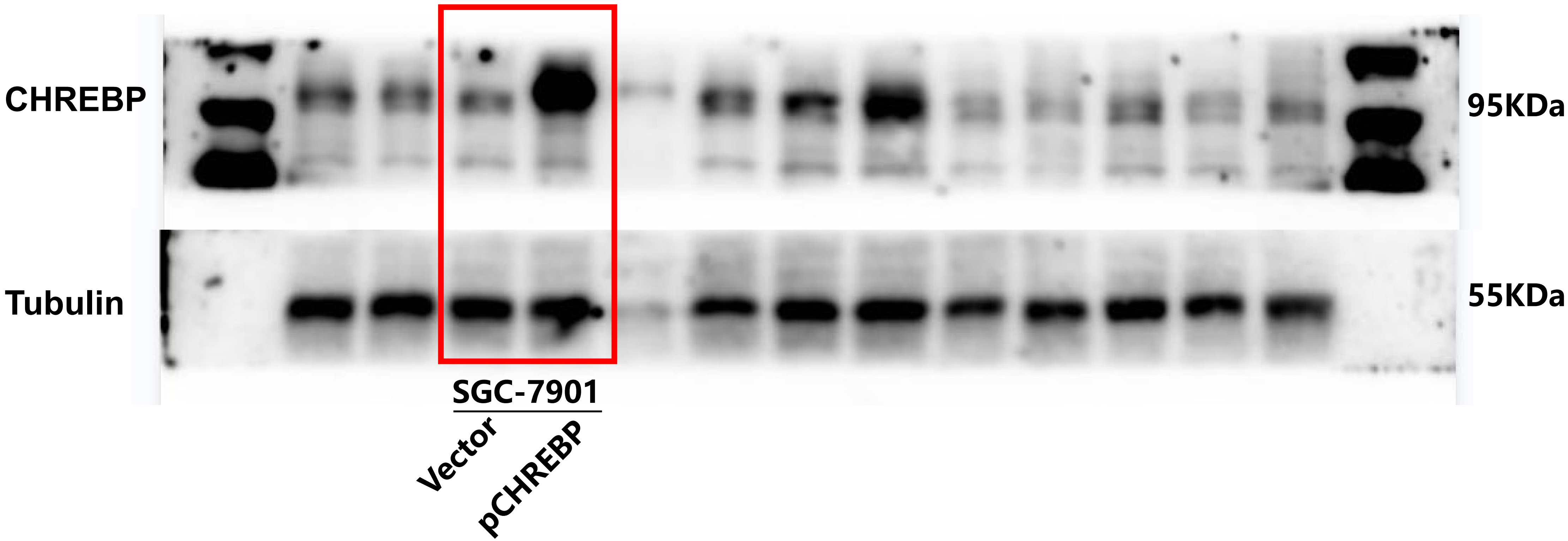
CHREBP
Tubulin
95KDa
55KDa
SGC-7901
Vector
pCHREBP

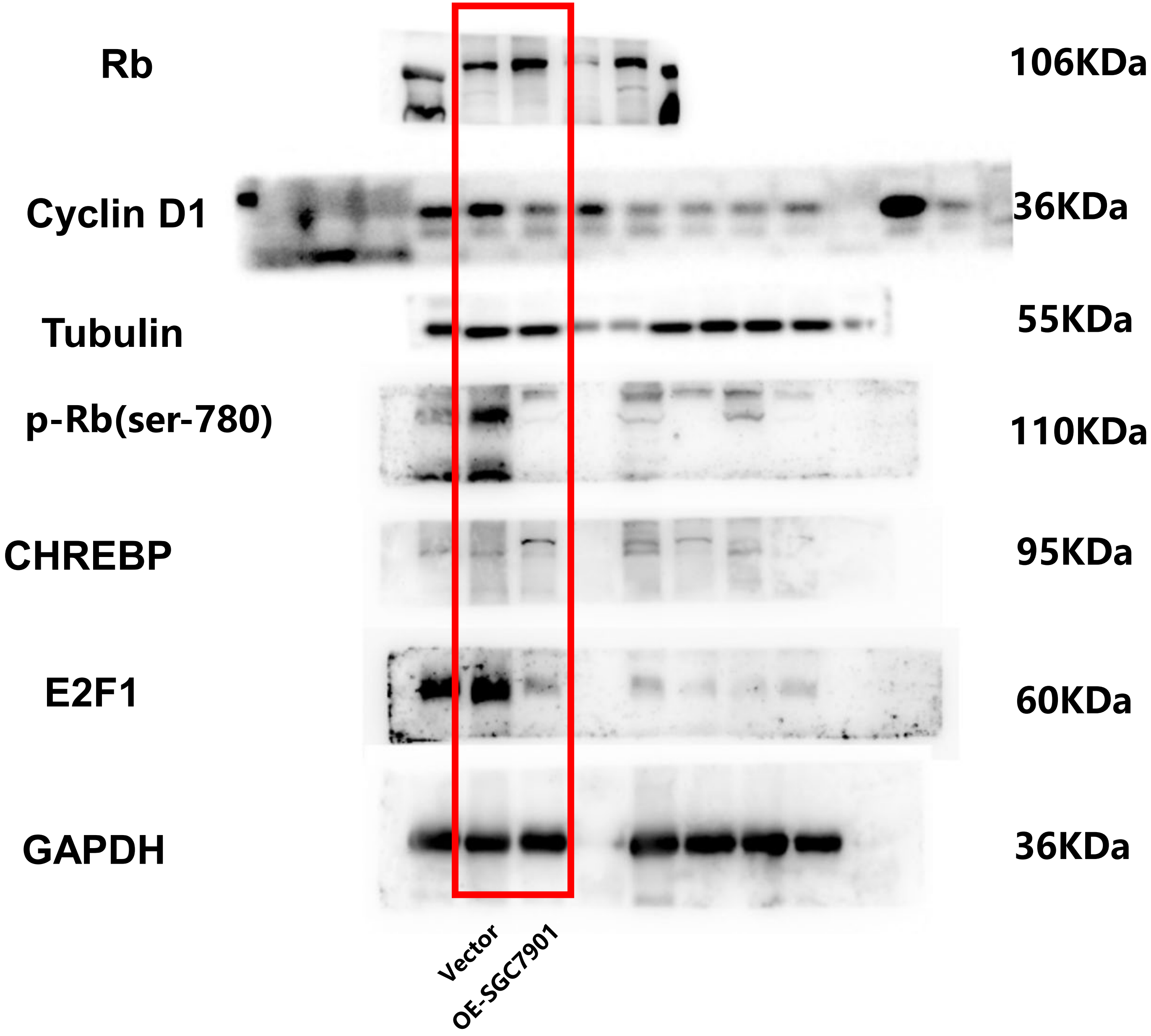
Rb
106KDa
Cyclin D1
36KDa
Tubulin
55KDa
p-Rb(ser-780)
110KDa
CHREBP
95KDa
E2F1
60KDa
GAPDH
36KDa
Vector
OE-SGC7901

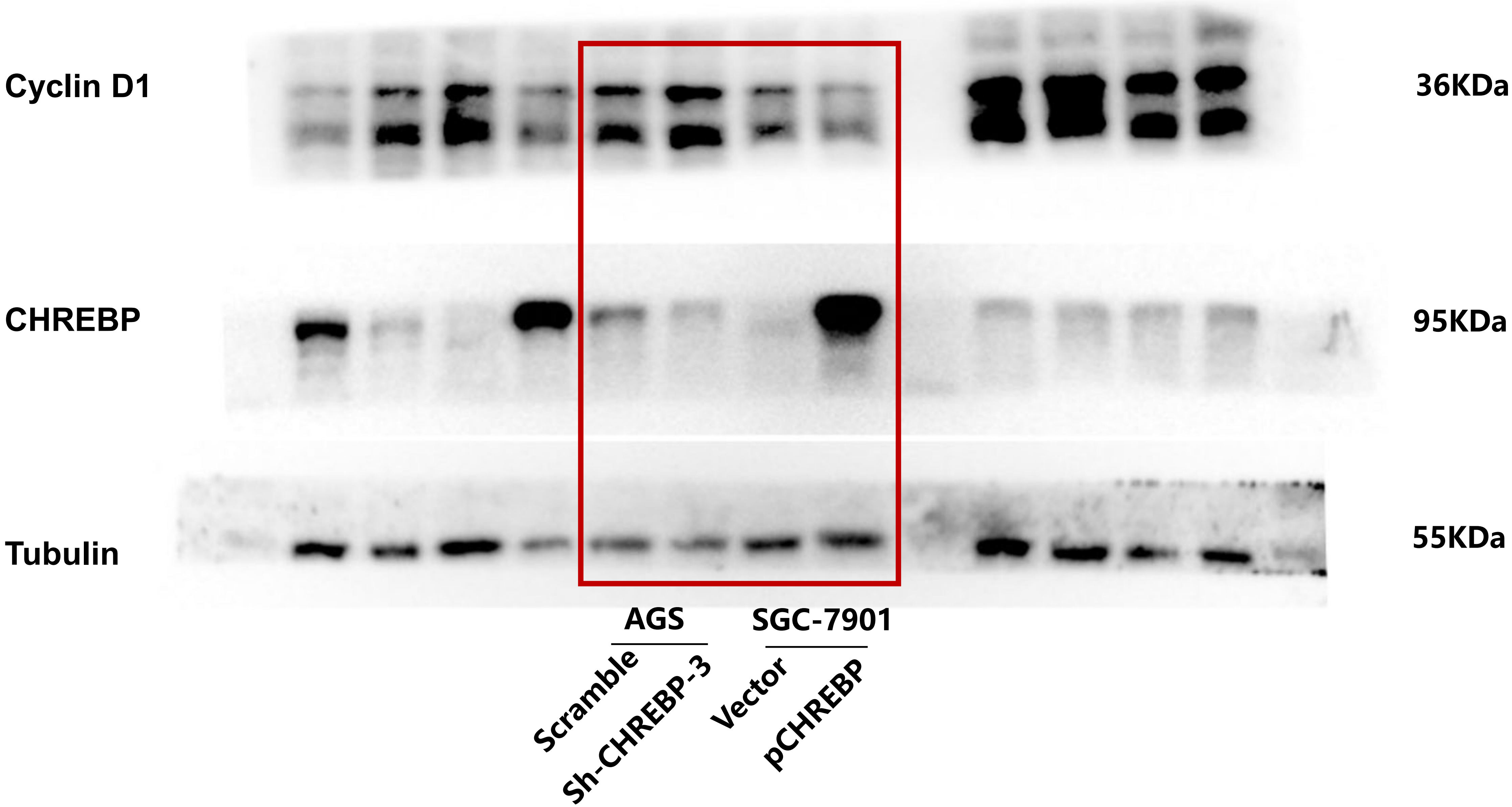

Cyclin D1
CHREBP
Tubulin
36KDa
95KDa
55KDa
AGS
SGC-7901
Scramble
Sh-CHREBP-3
Vector
pCHREBP

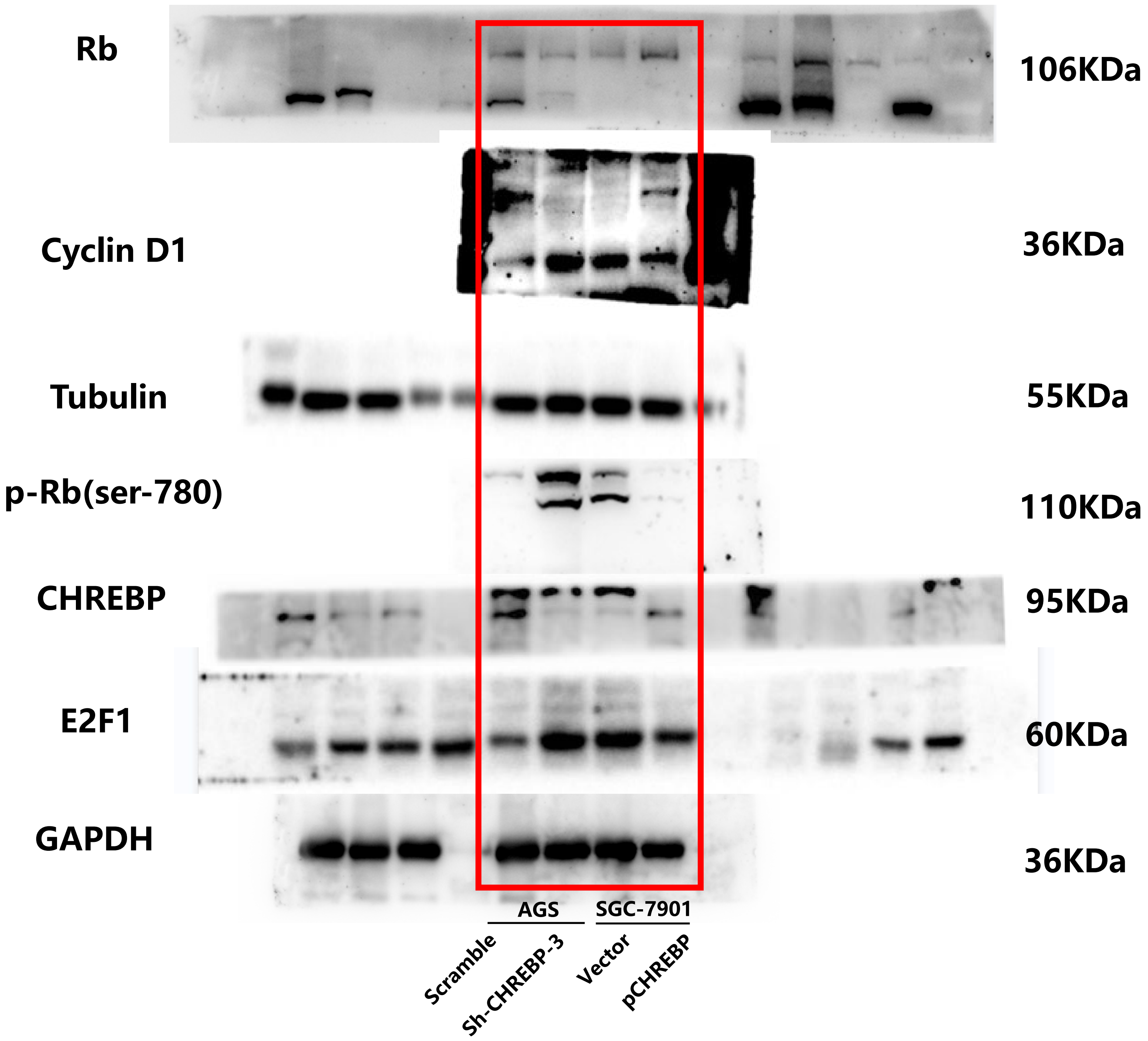
Rb
106KDa
Cyclin D1
36KDa
Tubulin
55KDa
p-Rb(ser-780)
110KDa
CHREBP
95KDa
E2F1
60KDa
GAPDH
36KDa
AGS
SGC-7901
Scramble
Sh-CHREBP-3
Vector
pCHREBP

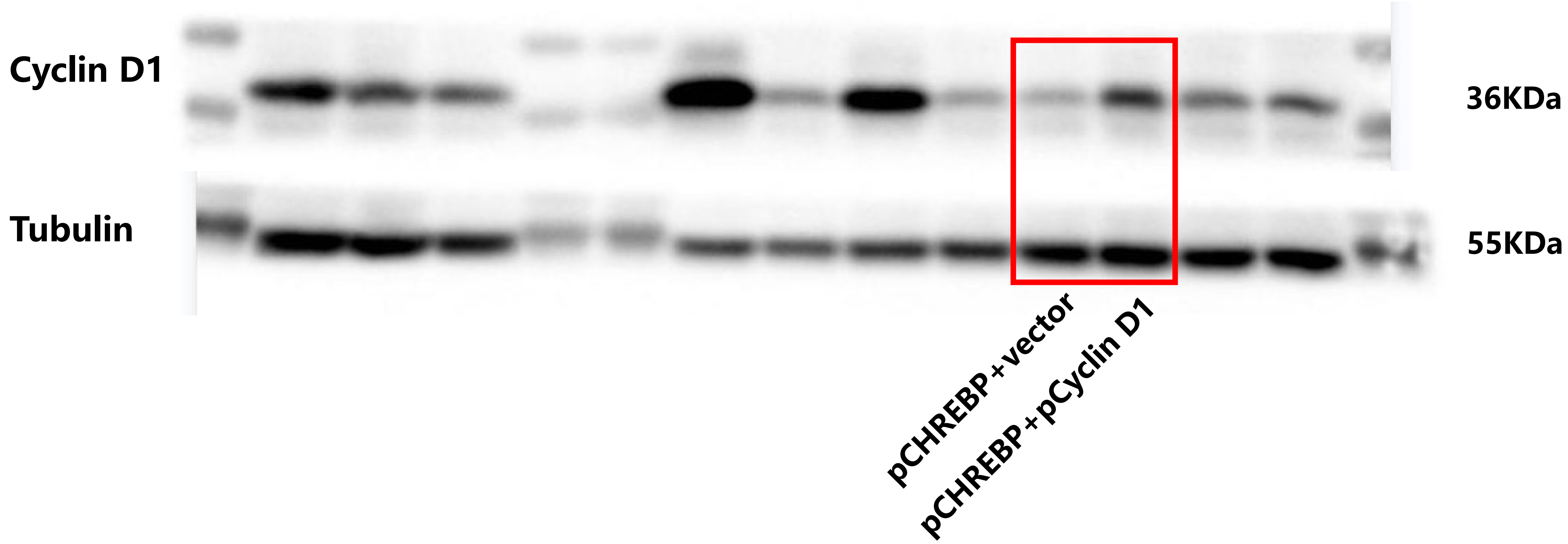
Cyclin D1
Tubulin
36KDa
55KDa
pCHREBP+vector
pCHREBP+pCyclin D1
