# Supplementary figures and images for: CHREBP suppresses gastric cancer progression via the cyclin D1-Rb-E2F1 pathway
Source: Cell Death Discov. 2022 Jun 29;8:300. doi: 10.1038/s41420-022-01079-1 (PMC9243070; doi:10.1038/s41420-022-01079-1)

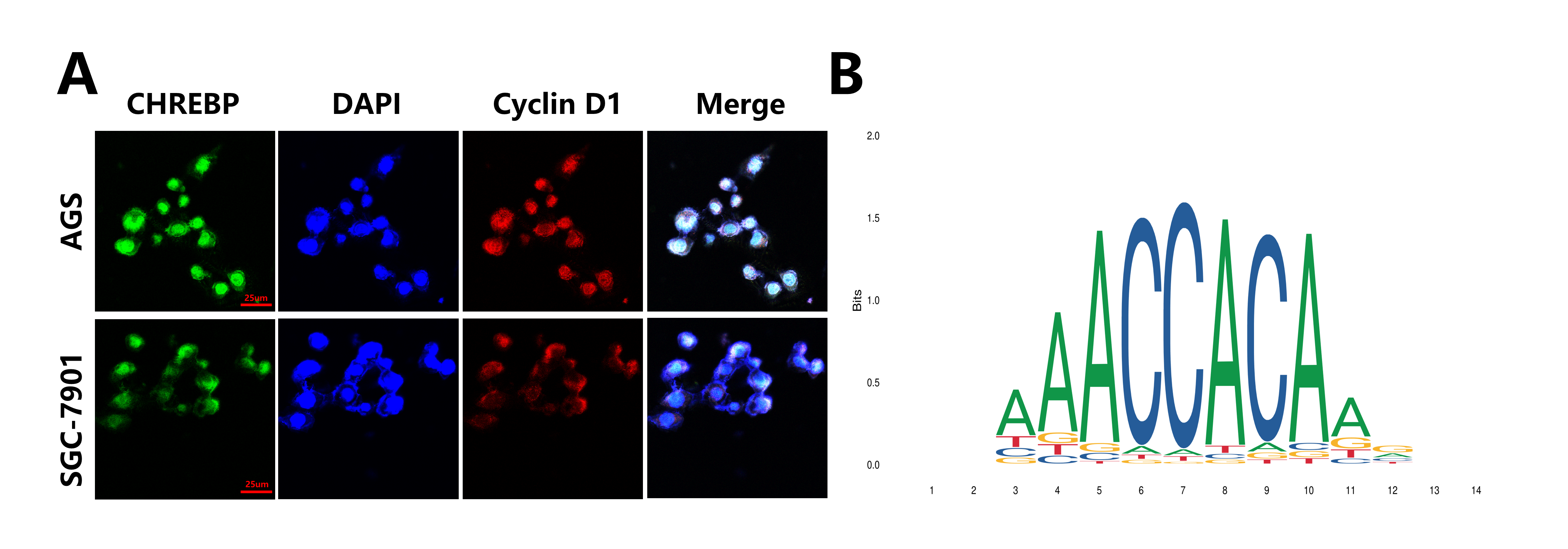

Supplement: Supplementary file 3 — Figure S1 [file 41420_2022_1079_MOESM3_ESM.tif]

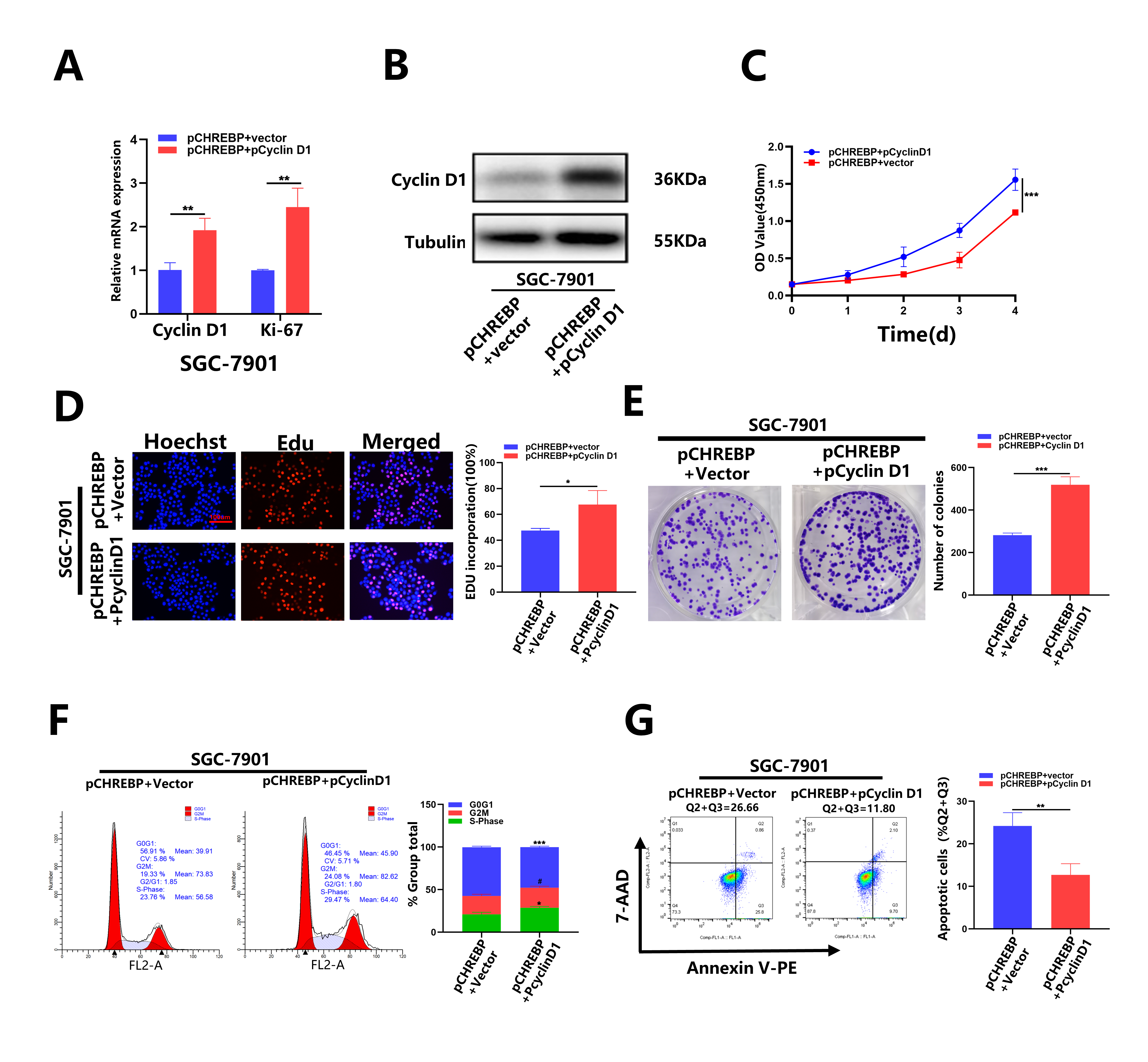

Supplement: Supplementary file 4 — Figure S2 [file 41420_2022_1079_MOESM4_ESM.tif]
